# Identification of Pacemaker Lead Position Using Fluoroscopy to Avoid Significant Tricuspid Regurgitation

**DOI:** 10.3390/jcm12144782

**Published:** 2023-07-19

**Authors:** Dicky A. Hanafy, Amiliana M. Soesanto, Budhi Setianto, Suzanna Immanuel, Sunu B. Raharjo, Herqutanto, Muzakkir Amir, Yoga Yuniadi

**Affiliations:** 1 Department of Cardiology and Vascular Medicine, Faculty of Medicine, Universitas Indonesia, National Cardiovascular Center Harapan Kita, Jakarta 11420, Indonesia; drdhanafy@yahoo.de (D.A.H.); amiliana14@gmail.com (A.M.S.); heybudhi@gmail.com (B.S.); sunu.b.raharjo@gmail.com (S.B.R.); 2 Department of Clinical Pathology, Faculty of Medicine, Universitas Indonesia, Dr. Cipto Mangunkusumo National Central Public Hospital, Jakarta 10430, Indonesia; suzanna.immanuel@gmail.com; 3 Department of Community Medicine, Faculty of Medicine, Universitas Indonesia, Jakarta 12345, Indonesia; hqtanto@gmail.com; 4 Department of Cardiology and Vascular Medicine, Faculty of Medicine, Universitas Hasanuddin, Dr. Wahidin Sudirohusodo Cardiovascular Center, Makassar 90245, Indonesia; dr.muzakkir@gmail.com

**Keywords:** fluoroscopy, jet area, lead impingement, transthoracic echocardiogram, tricuspid regurgitation

## Abstract

Permanent pacemaker implantation improves survival but can cause tricuspid valve dysfunction in the form of tricuspid regurgitation (TR). The dominant mechanism of pacemaker-mediated TR is lead impingement. This study evaluated the association between the location of the pacemaker leads crossing the tricuspid valve and the incidence of worsening TR and lead impingement using fluoroscopy. Lead positions were evaluated using perpendicular right anterior oblique (RAO) and parallel left anterior oblique (LAO) fluoroscopic angulation views of the tricuspid annulus. A two-dimensional transthoracic echocardiogram (TTE) was performed to evaluate the maximum TR jet area-to-right atrium ratio and define regurgitation severity. A three-dimensional TTE was performed to evaluate lead impingement. A worsening of TR was observed in 23 of 82 subjects. Most leads had an inferior position in the RAO view and a septal position in the LAO view. The mid position in the RAO view and septal position in the LAO view were risk factors for lead impingement. Mid and septal positions were associated with higher risks of significant TR and lead impingement. Lead impingement was associated with a high risk of significant TR. Pacemaker-mediated TR remains a significant problem after lead implantation.

## 1. Introduction

Permanent pacemaker (PPM) implantation is associated with undesirable effects on the tricuspid valve structure and function. Structural effects include valve damage during lead placement, mechanical interruption of normal leaflet coaptation, leaflet entrapment, entanglement of the subvalvular support structure, and endocarditis [1]. Functional effects include valvular stenosis and tricuspid valve regurgitation (TR). The right ventricle pacemaker lead crossing the tricuspid valve may cause fibrosis and thickening of the leaflets, thus impairing valve mobility and coaptation. Current pacemaker lead implantation procedures are guided by fluoroscopy and do not allow an evaluation of the tricuspid valve structure or function after the intracardiac leads have been implanted.

Moderate and severe TR based on echocardiography findings are classified as significant TR [2]. PPM implantation with the use of an RV lead crossing the tricuspid valve is associated with a two-fold higher risk of significant TR [3]. Pacemaker-mediated TR (PMTR) occurs when the pacemaker leads cause damage to the valve leaflets. It can also occur secondary due to RV dilatation and dysfunction caused by chronic dyssynchronous RV pacing [4]. A previous observational study showed that TR occurred when implanted device leads interfered with the normal tricuspid valve leaflet motion as viewed during a three-dimensional (3D) transthoracic echocardiogram (TTE); however, an association between the device lead position and TR incidence was not observed [5]. Another study noted that pacemaker lead positions on the annulus or inter-leaflet cleft that interfere with valvular mechanics can result in PMTR [3]. The position of pacemaker leads in the tricuspid valve can be identified using fluoroscopy, echocardiography, computed tomography, and magnetic resonance imaging.

The mechanisms of pacemaker-mediated TR are relatively unknown. They may include mechanical interference with tricuspid valve movement resulting in impingement, fibrosis, and excessive scarring. Inflammation and tissue fibrosis as well as scar formation involving valve tissues have been reported with PMTR [6,7].

Lin et al. reported that pacemaker lead impingement caused TR in 39% of 41 subjects; however, it was not associated with PPM lead positions [8]. Because PMTR is largely asymptomatic initially, its prevalence and relationship with PPM lead positions are understudied. Our study aimed to identify the positions of the pacemaker lead that are associated with significant TR.

## 2. Materials and Methods

This retrospective cohort study was conducted at the Harapan Kita National Cardiovascular Centre in Jakarta, Indonesia, from June 2020 to April 2021. The study population consisted of patients who underwent PPM implantation and were recruited consecutively from the medical record database and provided informed consent. Patients’ echocardiography was assessed at baseline and after implantation. Patients underwent noninvasive fluoroscopy assessment of pacemaker lead position. Inclusion criteria were as follows: age older than 18 years; underwent routine follow-up after PPM implantation; willing to participate in the study; and no previous right ventricular dysfunction (tricuspid annular plane systolic excursion [TAPSE] > 1.6 cm). The exclusion criteria were as follows: congenital or primary tricuspid disorder; left cardiac dysfunction with ejection fraction < 35%; pulmonary hypertension as defined by ESC/ERS Guidelines [9]; body constitution complicating echocardiography; more than one lead crossing the tricuspid valve; and rheumatic heart disease. This study did not include patients with permanent pacemakers with a single atrial lead, implantable cardioverter defibrillators (ICDs), or cardiac resynchronization therapy (CRT) devices. Information on patient comorbidities (coronary heart disease, hypertension, diabetes mellitus, atrial fibrillation) was acquired from the electronic medical record. Patients with heart failure included those with reduced or preserved ejection fraction. The study was approved by the Medical Research Ethic Committee of Universitas Indonesia Medical School in Jakarta, Indonesia. We did not conduct any special intervention or experiment; subjects underwent a non-invasive fluoroscopy examination and transthoracic echocardiography.

### 2.1. Fluoroscopy

Fluoroscopy angulation was performed to obtain en face and perpendicular in-plane views to evaluate the position of the pacemaker lead crossing the tricuspid valve annulus. Evaluation of the pacemaker lead position was viewed using the right anterior oblique (RAO) and left anterior oblique (LAO) projections. In the RAO position, the C-arm was angulated 30°; then, the annulus, which was seen as more radiolucent than the surrounding area, was evaluated. The radiolucent part was expected to be perpendicular when adjusting the RAO angulation by 30° to 40°, and the thinnest part was considered the most perpendicular annulus. We recorded the 3 s or three-heartbeat motion image (cineangiography) with RAO angulation. With LAO angulation, the C-arm was rotated 55° and 12° caudally to achieve a parallel en face tricuspid valve view; then, we recorded the 3 s or three-heartbeat motion image (cineangiography).

**Figure 1:**
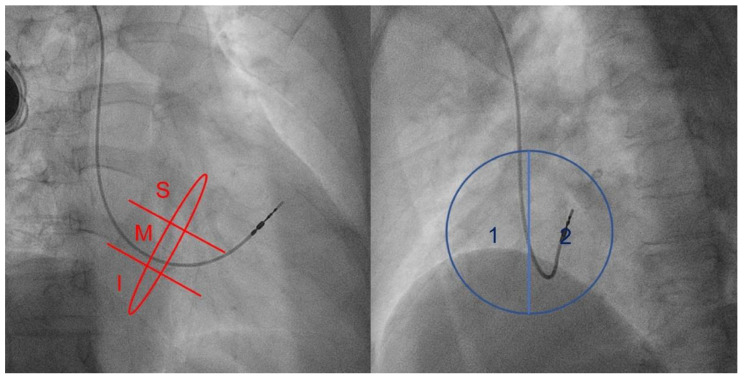
Mid-septal lead positions in the right anterior oblique (RAO) and left anterior oblique (LAO) views. S, superior; M, middle; I, inferior; 1, lateral position; 2, septal position.

In the RAO view, the pacemaker lead position was categorized into three locations, superior (S) mid (M), and inferior (I), in the annulus radiolucent position. In the LAO view, the pacemaker lead position was classified as lateral (1) or septal (2) (Figure 1).

### 2.2. Transthoracic Echocardiogram

**Figure 2:**
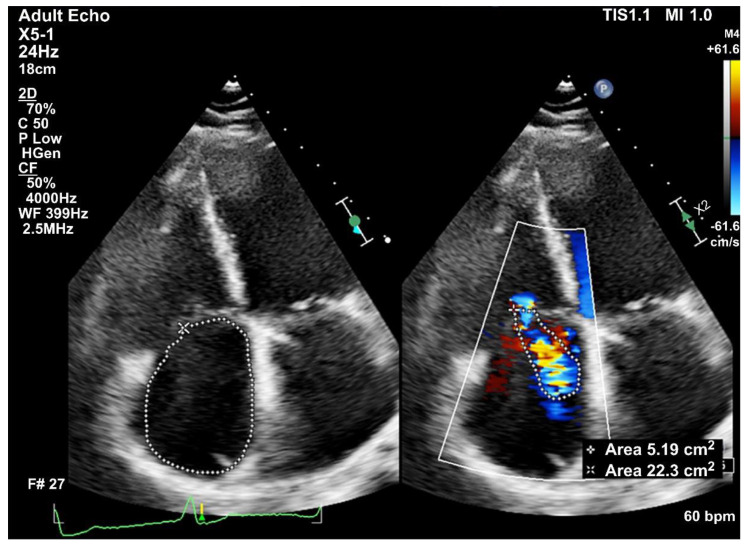
Tricuspid regurgitation (TR) jet area-to-right atrium ratio in the end-systolic phase measured using two-dimensional transthoracic echocardiography. The left panel shows the right atrium area (dot line = 22.3 cm^2^). The right panel shows the TR jet area (dot line = 5.19 cm^2^). The TR jet area-to-right atrium ratio = 23.3% (>20% is considered significant TR).

Trans-thoracic echocardiography (TTE) was performed before and after pacemaker implantation. Two-dimensional and 3D TTEs were performed by independent echocardiographers using GE Vivid E9 (GE Vingmed Ultrasound AS, Horten, Norway), GE Vivid E95 (GE Vingmed Ultrasound AS, Horten, Norway), and Philips Epiq Cvx (Philips Ultrasound, Bothell, WA, USA) with standard views: parasternal long and sort axis, apical, and subcostal views with M-mode, 2-dimensional echocardiography, and color Doppler ultrasonography. Two-dimensional TTE measured echocardiographic standard parameters such as the left ventricular internal dimension in diastole, left ventricular internal dimension in systole, ejection fraction measured by the Simpsons method, right ventricle dimensions, left atrium dimensions, vena contracta (VC), and regurgitation severity by comparing the maximum TR jet area with the right atrium area. Tricuspid annular plane systolic excursion (TAPSE) was measured on M-mode recordings of the lateral tricuspid annulus in an RV-focused view. The severity of tricuspid regurgitation was graded on a 5-point scale; 0 = no, 1 = trace, 2 = mild, 3 = moderate, and 4 = severe. Significant TR was defined as: moderate to severe TR as defined by the recommended parameters of the American Society of Echocardiography, with jet area-to-right atrium ratio ≥ 20% and VC ≥ 0.3 cm [10]. TR was not significant if the TR jet area-to-right atrium ratio < 20% and VC < 0.3 cm or mild based on the recommended parameters of the American Society of Echocardiography guidelines (Figure 2) [10].

**Figure 3:**
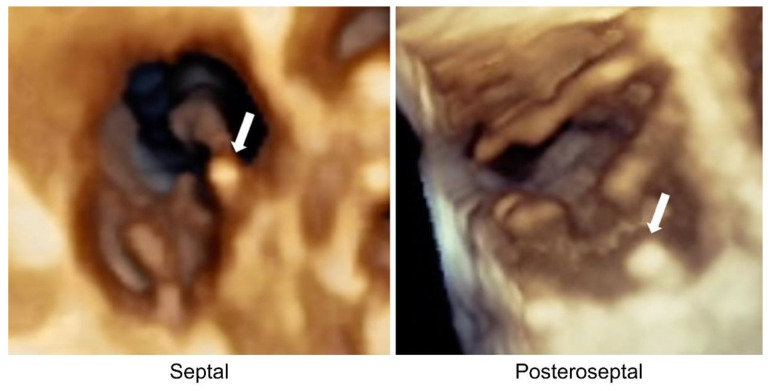
Pacemaker lead position viewed with three-dimensional (3D) transthoracic echocardiography. From left-to-right: (1) pacemaker lead observed as impinging on the septal leaflet; and (2) pacemaker lead observed in the posteroseptal commissure. Arrows point to the pacemaker lead.

The 3D TTE was performed using the standard position and en face view. We evaluated the tricuspid valve morphology, pacemaker lead position, and lead impingement. Lead impingement on the anterior, posterior, and septal leaflets of the tricuspid valve was defined as impinging; however, a pacemaker lead located in the anteroposterior, anteroseptal, and posteroseptal commissures or with a central position (in the middle of coaptation and with no impingement on the tricuspid leaflet) was defined as non-impinging (Figure 3). The echocardiographer was blind to the fluoroscopy results of the lead position.

### 2.3. Study Variables

The dependent variables were the presence of worsening to significant TR as defined. Patients who had the same degree of TR (non-significant or significant TR) at baseline and did not progress to a worse degree of TR post-implantation were defined as non-worsening TR. The independent variables were the pacemaker lead position (evaluated by fluoroscopy) and lead impingement on the tricuspid valve (evaluated by 3D TTE).

### 2.4. Statistical Analysis

Data were expressed as mean ± standard deviation (if normally distributed) or median ± interquartile range (if not normally distributed). The normality of the distribution of variables was assessed using the Kolmogorov–Smirnov test. Associations between two categorical variables were evaluated using the chi-square test or Fisher’s test. Mean differences between two groups were analyzed with the unpaired *t*-test (if normally distributed) or Mann–Whitney test (if not normally distributed). A two-tailed *p* < 0.05 was considered statistically significant. A multivariate analysis was performed for significant variables, and a logistic regression model was used to identify the risk factors for TR. Data were processed using SPSS version 25.0 software (IBM Corp., Armonk, NY, USA).

## 3. Results

**Figure 4:**
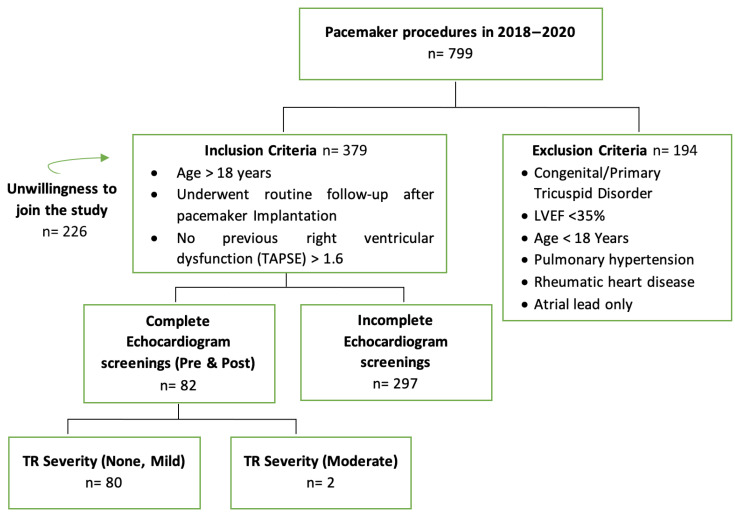
Flow diagram of patients receiving a pacemaker included in the study.

The study included a final sample size of 80 subjects with a mean age of 61.3 ± 12.27 years; 58.5% females). During 2018 until the end of 2020, there were 799 patients who were implanted with a pacemaker (Figure 4) A significant number of patients with sinus node dysfunction were implanted with atrial single chamber pacemaker due to cost, which were excluded from this study. There was also a significant number of patients who did not have an echocardiogram before pacemaker implantation at our center but mostly at the referring centers without detailed description. Lastly, a significant number of patients had no follow up echo because these patients were referred back to the referring centers or refused to participate in the study. Hence no 3D echo could be performed mostly because of the distance to our center and the health referral system in our country.

The indications for pacemaker implantation were complete heart block (57.3%), sinus node dysfunction (29.3%), or other AV nodal conduction disturbance (13.4%). Other AV nodal conduction disturbances were defined as incomplete heart block either type 2 s degree AV block or trifascicular block. Most patients had a dual-chamber pacemaker (72.0%). The median pacing percentage was 100%. The patients had a mean implantation duration of 35.22 months (standard deviation 35.81 months). At baseline, non-significant TR was noted in 80 (95.6%) patients, and moderate to severe TR was noted in 2 (2.4%) patients. Of the patients with non-significant TR at baseline, the TR remained non-significant in 57 (71.25%) patients at the time of the study. TR worsened to significant TR in 23 (26.25%) patients. In one patient, TR improved and became non-significant. Hence, worsening TR was present in a significant proportion of patients (Table 1). There were no differences in RA and RV dimensions, TAPSE, or RV fractional area change (FAC). The patients with worsening TR were, on average, older and had a longer mean duration of pacemaker implantation but these differences were not statistically significant. Age, sex, hypertension, coronary heart disease, atrial fibrillation, and heart failure were clinical factors associated with a worsening TR.

Most pacemaker leads had an inferior position (63.75%) in the RAO view and a septal position (77.5%) in the LAO view. A mid position in the RAO view and septal position in the LAO view were risk factors for worsening TR. A mid-septal position was associated with the highest risk of worsening TR (Table 2). Lead impingement was found in 26.25% of subjects. A fluoroscopic mid position in the RAO view increased the risk of lead impingement (*p* < 0.001). A septal position in the LAO view increased the risk of lead impingement, but this was not statistically significant. Lead impingement was most prevalent in the mid-septal position in the combined RAO–LAO view (Table 3). Further analysis showed that lead impingement was associated with a high risk of significant TR (*p* < 0.001) (Table 4).

Based on univariate analysis, there were several variables that fulfilled the requirements for multivariate analysis namely age, sex, hypertension, atrial fibrillation, presence of heart failure, implant duration, mid fluoroscopy position on RAO view, septal fluoroscopy position on LAO, and mid-septal fluoroscopy position in the combined RAO–LAO view. The logistic regression analysis was performed using the backward stepwise elimination method to obtain a final model that had statistical significance. The multivariate analysis showed that a fluoroscopic mid-septal location was a significant predictor of worsening TR (*p* = 0.001; odds ratio [OR], 8.352; 95% confidence interval [CI], 2.514–27.746) In the final model, hypertension and atrial fibrillation were also included as clinical risk factors that were analyzed, of which hypertension was considered a significant risk factor (Table 5).

## 4. Discussion

### 4.1. Risk Factors for Significant TR

Our study reported a 28.75% progression to significant TR; although this may be considered high, it is within the range of the previously reported incidences of 18.3%, 24.2%, and 39% [11,12,13]. In our cohort, there were no differences in RA and RV dimensions, TAPSE, and also RV fractional area change (FAC) between those with and without worsening of TR.

The clinical factors associated with worsening TR that were evaluated in our study were age, sex, history of coronary heart disease, hypertension, atrial fibrillation, presence of heart failure, and duration of implantation. The patients with significant TR had a higher mean age, consistent with a previously reported finding that an age between 72 and 75 years was a risk factor for TR [12].

Our study showed a higher prevalence of significant TR for women, but the difference was not statistically significant. Riesenhuber et al. [14] reported an increase in the prevalence of significant TR for women, but this difference might have been caused by differing sample sizes. However, female sex could still be considered a risk factor for TR after PPM implantation.

A history of coronary heart disease was not associated with significant TR. A similar result was reported by another study that found a lower prevalence of significant TR for patients with coronary heart disease; however, this finding was not statistically significant [15].

Hypertension was a predictor of significant TR, and we found a statistically significant association in our study (*p* = 0.026). The mechanism of this in our subjects is unclear since all patients with pulmonary hypertension were excluded. Chronic hypertension could increase left ventricle load resulting in hypertensive cardiac disease, which, if progressive, can develop to left heart failure. Further backflow from the left ventricle to the left atrium may result in diastolic dysfunction, mitral regurgitation, or decreased left atrial compliance. Increased pulmonary arterial hypertension will worsen pulmonary vessel remodeling and can increase the right ventricle load, possibly leading to right ventricle dilatation. At this stage, the risk of TR increases [16,17].

Atrial fibrillation may result in TR in the long term because of right atrial dilatation. Enlargement of the right atrium is followed by annular dilatation [18]. Previous studies showed that TR in the elderly with chronic atrial fibrillation was secondary to right atrial enlargement, right ventricle dysfunction, and tricuspid annulus dilatation, regardless of pulmonary hypertension [19,20]. Although statistically not significant, atrial fibrillation was found in almost 35% of the cohort with worsening TR, compared to only 20% in the cohort without worsening TR. There were no differences in the atrial dimensions (RA area, LA dimension, and LAVI) in both groups.

Our study showed that several lead positions, as identified by fluoroscopy, were risk factors for lead impingement. It is also known that TR is associated with reduced survival rates. Because impingement disturbs tricuspid valve motion and results in significant TR, this finding may predispose patients to right heart failure. With the expanding use and indications of cardiac implantable electronic devices such as pacemakers in bradyarrhythmias, it is important to recognize the remodeling and enlargement of the right heart chambers, which induces further annular dilatation and creating a never-ending cycle of right heart dilatation and worsening TR. A study by Vaturi et al. noted that active right ventricular pacing may increase the severity of TR; the median pacing rate was 100% in the current population [21]. In the present study, right ventricular dyssynchrony as measured by the percentage of right ventricular pacing was not associated with significant TR.

### 4.2. Association between the Pacemaker Lead Position and Worsening TR

Some mechanisms postulated include perforation, laceration, lead entanglement, or fibrous adherence, thus impairing valve mobility and coaptation [8,11,22,23,24]. Lead impingement on valve leaflets can also result in mechanical disruption of the valve closure. The pacemaker lead position was evaluated in the RAO view with a 30° angulation and in the LAO view with a 55° angulation using fluoroscopy. Our study showed that certain lead positions significantly increased the prevalence of worsening TR (*p* < 0.001). The highest prevalence of worsening TR was observed in the mid-septal position, and the most prevalent position of leads in all patients was inferior-septal. In the mid-lateral position, we did not observe any significant TR.

A previous study by Poorzand et al. concluded that defining lead positions with fluoroscopy could not predict increased TR severity (*p* > 0.05). In this study, they only used a single antero-posterior view for fluoroscopy without further angulation [25]. In contrast, our study showed that lead positions defined by fluoroscopy using certain angulations could predict significant TR. A mid-septal position increased significant TR; hence, our findings suggest that mid-septal positions should be avoided when placing leads.

### 4.3. Association between the Pacemaker Lead Position and Lead Impingement Status

There was a significant association between the lead position defined by fluoroscopy and lead impingement as identified by echocardiography. The mid position in the RAO view was a risk factor for lead impingement, as was the septal position in the LAO view. The most prevalent lead impingement was found in the mid-septal position. A study by Addetia et al. showed that the presence of a device lead interfering with normal tricuspid valve leaflet motion was the most significant factor associated with the development of TR after device placement, thus supporting our findings on the impact of lead impingement on significant TR [26].

To reduce the risk of PMTR, it should be ensured that the non-mid-septal position provides enough slack for the pacemaker lead during implantation, thus allowing the lead to move inferiorly. This could be performed using the RAO view with 30° of angulation. The posteroseptal commissure should be the goal of lead placement because TR and lead impingement risks are low when the lead is positioned inside the commissure. When the lead has enough slack to move to a more inferior position, the LAO view with 55° of angulation is useful for ensuring that the lead is placed more septally. It is preferrable to position the lead in the posteroseptal commissure of the tricuspid valve to avoid the mid-septal position. This position should be easier to achieve than the mid-lateral and inferior-lateral positions. In the future, it would be beneficial to implant the device leads using 3D TTE guidance.

### 4.4. Study Limitations

One of the limitations of this study is that our center is a tertiary referral care center, in which patients who underwent echocardiography were more likely to develop symptoms after implantation or other forms of valvular or myocardial dysfunction. Echocardiographic measurements were also not performed in an independent echocardiographic laboratory and interobserver differences regarding the assessment of TR severity may have influenced the results. Other limitations of this study were the relatively small sample size and the various timelines of patient evaluation, which needs to be improved in future studies.

## 5. Conclusions

We confirmed that PMTR remains a significant problem after pacemaker lead implantation. Only hypertension was found to be a clinical risk factor associated with significant TR. Pacemaker lead positions are adequately visualized by fluoroscopy in the RAO and LAO views. Leads in the mid and septal positions were associated with higher risks of worsening TR and lead impingement. Three-dimensional TTE remains an invaluable tool for assessing tricuspid valve function and may be used to guide future intracardiac device lead implantation procedures.

## Figures and Tables

**Table 1:** Baseline characteristics.

Characteristics	Tricuspid Regurgitation		OR (95% CI)	*p*-Value
	Worsening TR (*n* = 23)	Not Worsening TR (*n* = 57)		
Sex, *n* (%)				
Male	7 (30.4)	25 (43.9)	0.56 (0.20–1.57)	0.267 *
Female	16 (69.6)	32 (56.1)		
Mean age, years	63.35 ± 11.24	59.93 ± 12.70		0.221 †
History of coronary heart disease				
Yes	9 (39.1)	11 (19.3)	2.69 (0.93–7.80)	0.064 *
No	14 (60.9)	46 (80.7)		
Hypertension				
Yes	20 (87.0)	35 (61.4)	4.19 (1.11–15.77)	0.026 *
No	3 (13.0)	22 (38.6)		
Diabetes mellitus, type 2				
Yes	7 (30.4)	21 (35.6)	0.75 (0.27– 2.12)	0.587 *
No	16 (69.6)	36 (63.2)		
Atrial fibrillation				
Yes	8 (34.8)	11 (19.3)	2.23 (0.76–6.58)	0.141 *
No	15 (65.2)	46 (80.7)		
History of heart failure				
Yes	10 (43.5)	14 (24.6)	2.36 (0.85–6.56)	0.095 *
No	13 (56.5)	43 (75.4)		
Mean PPM implantation duration, months/median	43.91 ± 40.52/32 (7–151)	32.51 ± 33.96/24 (8–179)		0.271 †
Median pacing percentage	100.0 (2.1–100.0)	100.0 (0.2–100.0)		0.591 †
PPM indication				
Sinus node dysfunction	7 (30.4)	16 (28.1)		0.706
Complete heart block	14 (60.9)	32 (56.1)		
Other AV conduction disturbance	2 (8.7)	9 (15.8)		
PPM type				
Single chamber	8 (34.8%)	14 (24.6%)	1.64 (0.57–4.67)	0.354 *
Dual chamber	15 (65.2%)	43 (75.4%)		
Echocardiography				
LVEF, %	61.86 ± 14.00	65.48 ± 10.87		0.257 ‡
LVIDd, mm	48.49 ± 5.80	47.05 ± 8.95		0.481 ‡
LVIDs, mm	30.46 ± 7.87	28.57 ± 7.86		0.395 †
LAVI, mL/mm^2^	40.57 ± 17.13	36.12 ± 12.55		0.233 †
Left atrial dimension, mm	39.72 ± 7.03	37.90 ± 6.98		0.305 †
TAPSE, mm	21.67 ± 4.07	22.56 ± 4.10		0.324 †
RA Area, cm^2^	17.70 ± 4.90	15.78 ± 3.32		0.068 ‡
RVd basal, cm	3.59 ± 0.70	3.40 ± 0.49		0.280 ‡
RVd mid, cm	2.42 ± 0.47	2.39 ± 0.43		0.848 †
RVd long, cm	6.24 ± 0.82	6.37 ± 0.70		0.496 ‡
FAC, %	43.32 ± 7.14	45.39 ± 6.38		0.253 ‡

TR, tricuspid regurgitation; PPM, permanent pacemaker; AV, atrioventricular; LVEF, left ventricular ejection fraction; LVIDd, left ventricular internal dimension in diastole; LAVI, left atrial volume index; TAPSE, tricuspid annular plane systolic excursion; LVIDs, left ventricular internal dimension in systole; RA, right atrium; RVd basal, right ventricular basal diameter at end-diastole; RVd mid, right ventricular mid diameter at end-diastole; RVd long, right ventricular longitudinal diameter at end-diastole; FAC, fractional area change; OR, odds ratio; 95% CI, 95% confidence interval. * Chi-square test. † Mann–Whitney test. ‡ Unpaired *t*-test.

**Table 2:** Association between the pacemaker lead position and significant tricuspid regurgitation (*n* = 80).

Fluoroscopy	Worsening TR (*n* = 23)	Not Worsening TR (*n* = 57)	OR (95% CI)	*p*-Value
RAO, *n* (%)				
Mid	14 (60.9)	15 (26.3)	4.36 (1.56–12.13)	**0.04**
Inferior	9 (39.1)	42 (73.7)		
LAO, *n* (%)				
Septal	21 (91.3)	41 (71.9)	4.10 (0.86–19.52)	**0.06**
Lateral	2 (8.7)	16 (28.1)		
RAO–LAO, *n* (%)				
Mid-lateral	0	6 (10.5)		**0.001**
Mid-septal	14 (60.9)	9 (15.8)		
Inferior-lateral	2 (8.7)	10 (17.5)		
Inferior-septal	7 (30.4)	32 (56.1)		
RAO–LAO, *n* (%)				
Mid-septal	14 (60.9)	9 (15.8)	8.30 (2.76–24.90)	**<0.001**
Others	9 (39.1)	48 (84.2)		

TR, tricuspid regurgitation; RAO, right anterior oblique; LAO, left anterior oblique; OR, odds ratio; 95% CI, 95% confidence interval. Bold values indicate significant results.

**Table 3:** Association between the pacemaker lead position and lead impingement (*n* = 80).

Fluoroscopy	Impinging (*n* = 21)	Non-Impinging (*n* = 59)	OR (95% CI)	*p*-Value
RAO, *n* (%)				
Mid	16 (76.2)	13 (22.0)	11.32 (3.49–36.77)	<0.001
Inferior	5 (23.8)	46 (78.0)		
LAO, *n* (%)				
Septal	19 (90.5)	43 (72.9)	3.54 (0.74–16.92)	0.097
Lateral	2 (9.5)	16 (27.1)		
RAO-LAO, *n* (%)				
Mid-Septal	16 (76.2)	7 (11.9)	23.77 (6.63–85.25)	<0.001
Other	5 (23.8)	52 (88.1)		

RAO, right anterior oblique; LAO, left anterior oblique; OR, odds ratio; 95% CI, 95% confidence interval.

**Table 4:** Association between lead impingement and significant tricuspid regurgitation prevalence (*n* = 80) (*p* < 0.001)**.**

Lead Impingement	Tricuspid Regurgitation		OR (95% CI)
	Worsening (*n* = 23)	Not Worsening (*n* = 57)	
Impinging, *n* (%)	18 (78.3)	3 (5.3)	64.8 (14.06–298.52)
Non-impinging, *n* (%)	5 (21.7)	54 (94.7)	

OR, odds ratio; 95% CI, 95% confidence interval.

**Table 5:** Multivariate analysis.

	Characteristic	β	SE	OR_adj_ (95% CI)	*p*-Value
	PPM lead position				
	Inferior-septal (ref)	-	-	-	
First Step	Mid-septal	2.558	0.735	12.913 (3.056–54.554)	**0.001**
	Sex	0.638	0.749	1.892 (0.436–8.212)	**0.038**
	Age (years)	−0.006	0.028	0.994 (9.942–1.049)	0.831
	Duration of implantation (months)	0.011	0.008	1.011 (0.005–1.027)	0.175
	Hypertension	1.871	0.903	6.493 (1.106–38.125)	**0.038**
	Atrial fibrillation	1.513	0.762	4.541 (1.021–20.207)	**0.047**
	Coronary heart disease	−1.415	0.850	0.243 (0.046–1.285)	0.096
	Heart failure	0.474	0.668	1.607 (0.434–5.951)	0.478
	Constant	−0.942			
	Hypertension	1.878	0.858	6.543 (1.218–35.140)	**0.029**
Final Step	Atrial fibrillation	1.105	0.640	3.019 (0.862–10.575)	0.084
	Mid-septal position	2.123	0.613	8.352 (2.514–27.746)	**0.001**
	Constant	−3.563			

β, logistic regression coefficient; SE, standard error; OR_adj_, adjusted odds ratio; CI, confidence interval; ref, reference; PPM, permanent pacemaker.

## Data Availability

The datasets used and/or analyzed during the current study are available from the corresponding author on reasonable request.
